# Electroanalytical Enzyme Biosensor Based on *Cordia superba* Enzyme Extract for the Detection of Phytomarkers in Kombucha

**DOI:** 10.3390/bios12121112

**Published:** 2022-12-01

**Authors:** Erica A. Batista, Marx O. A. Pereira, Isaac Y. L. Macêdo, Fabio B. Machado, Emily K. G. Moreno, Elgia P. Diniz, Italo G. V. Frazzão, Lorrayne S. C. Bernardes, Severino C. B. Oliveira, Eric S. Gil

**Affiliations:** 1 Faculdade de Farmácia (FF), Universidade Federal de Goiás (UFG), Goiânia 74605-170, Brazil; 2 Departamento de Química (DQ), Universidade Federal Rural de Pernambuco (UFRPE), Recife 52171-900, Brazil

**Keywords:** polyphenolxidase enzymes, kombucha, *Cordia superba*

## Abstract

Antioxidants are responsible for many beneficial health effects and are highly present in natural products, such as kombucha. Biosensors’ development targeting antioxidants and phytomarkers are an active research field. This work aimed to propose a voltammetric polyphenolxidase (*Cordia superba*) biosensor for catechin and total phenolic compounds quantification in kombucha samples. Optimizations were performed on the biosensor of *Cordia superba* to improve the accuracy and selectivity, such as enzyme–substrate interaction time, analytical responses for different patterns and signal differences with the carbon paste and modified carbon paste electrode. Kombucha probiotic drink samples were fermented for 7 to 14 days at a controlled temperature (28 ± 2 °C). A linear curve was made for catechin with a range of 10.00 to 60.00 µM, with a limit of detection of 0.13 µM and limit of quantification of 0.39 µM. The biosensor proposed in this work was efficient in determining the patterns of phenolic compounds in kombucha.

## 1. Introduction

Electrochemical methods are the gold option for investigating redox systems, and biosensors can maximize their applications by combining highly selective biorecognition systems with very sensitive transducer materials. These biosensors have been explored in different areas, including health, environmental and quality control [1,2].

Different polyphenol oxidase (PPO) electrochemical biosensors have been proposed for the detection and quantification of numerous phenolic compounds in different matrices [3,4,5,6]. These biosensors have advantages such as stability, selectivity and sensitivity [3,4,5,6].

PPOs are enzymes with an active site of metallic copper and are coordinated with amino acids, histidine, cysteine and methionine; they are found in different parts of plants, bacteria, animals and fungi [3,4,5,6]. The molecular mass of PPOs depends on the source and these enzymes can be classified as tyrosine (E.C.1.14.18.1), catechol oxidase (EC 1.10.3.1) and laccase (EC 1.10.3.2) [3,4,5,6,7]. PPOs catalyze the hydroxylation of a monophenol to a diphenol, following its oxidation to a quinone, by molecular oxygen [3,4,5,6,7].

*Cordia superba* (*CS*) is a Boraginaceae commonly known as aloe-white [8]. The family Boraginaceae is commonly found in tropical areas of Europe, Asia, Africa, Australia and the Americas. Species of *Cordia* are largely employed in folk medicine worldwide [9]. In the last decades, scientific studies of Cordia species have intensified, demonstrating the great interest in phytochemical, biological and pharmacological studies [10]. Additionally, the prospection of bioactive molecules of *Cordia* species is a promising source since the plant is characterized by easy obtainment and low cost [11,12].

Probiotic drinks have been gaining prominence around the world for their beneficial health effects. One example is the fermented drink kombucha based on green tea, which has, predominantly, flavonols and phenolic acids in its composition [12,13]. Such phytochemical compounds are recognized by antioxidant, antimutagenic and antimicrobial properties [3,12,13].

The total phenol determination can be achieved by spectrometric and electroanalytical methods, in which the differential pulse voltammetry (DPV) is highlighted to offer higher sensitivity, due to low capacitive current interferences. Moreover, the DPV pattern allows for underestimating the polyphenol profile, even in crude samples [3,4,5,6,7].

Thus, the main objective of this work was the development of a PPO electrochemical biosensor with carbon paste (CP) using the crude extract of *Cordia superba*. The experimental conditions for the preparation and applications of the proposed biosensor were optimized. The carbon paste biosensor with *Cordia superba* (*CS*) extract was then called CP50*CS* and applied for the detection and quantification of phenolic compounds in kombucha samples.

## 2. Materials and Methods

### 2.1. Chemicals and Reagents

*Cammelia sinensis* (tea plant), Symbiotic Cultures of Bacteria and Yeasts (SCOBYs) from German, American, Canadian and Jun SCOBY, and honey were obtained from local shops in Goiânia, Goiás, Brazil. All solutions were prepared using analytical grade reagents and double distilled Milli-Q water (conductivity 0.1 µS.cm^−1^) (Millipore S.A., Molsheim, France). Cafeic acid, catechin, catechol, gallic acid, epicatechin and rutin were purchased from Sigma Chemical Co. (St. Louis, MO, USA). All solution standards were prepared with a concentration of 100 µM from the dilution of stock solutions (1 mM). The carbon paste was prepared using graphite powder and mineral oil, both from Sigma-Aldrich (St. Louis, MO, USA).

### 2.2. Plant Material and Preparation of the Raw Vegetable Extract

*Cordia superba* (*CS*) plant material was collected from a single plant located in Goiania, Brazil in February 2022. Leaves, unripe and ripe fruits and twigs were washed with distilled water, packed in polyethylene bags and stored until analysis. To obtain the crude extract, a solution was prepared with 2 g of leaves, 2 g of greens or 2 g of ripe fruits, macerated in a pestle mortar and added to 10 mL of phosphate buffer saline (PBS) solution, pH 7.0, for 10 min. To obtain the crude extract from the branches, 2 g was weighed with sizes of approximately 5 cm and PBS solution was added for 10 min. The solution was homogenized and filtered through filter paper, resulting in the crude extract of *Cordia superba.*

### 2.3. Preparation of Kombucha (Fermented Tea)

The preparation of fermented kombucha tea was based on the methodology proposed by [14]. Organic green tea leaves were infused with 20% sucrose as a carbon source, for 5 to 10 min and then allowed to cool at room temperature. The sugar solution was transferred to a container covered with sterile gauze with the aid of an elastic band. After cooling the tea, the SCOBY, composed of Acetobacter sp. (Acetobacter xylinum, Acetobacter aceti, Acetobacter pasteurianus) and Brettanomyces sp. (Brettanomyces bruxellensis, Brettanomyces intermedius), was added and fermented for 7 to 14 days.

To reduce the risk of contamination by pathogenic and spoilage microorganisms, sanitary measures were adopted and 2 mL of the previously fermented culture broth was added to the solution, reducing the pH of the solution being kept in an oven at room temperature. After four days, a new Scoby film had formed, after seven days the film had already formed; the resulting beverage was filtered to remove the culture and remaining suspended microorganisms.

### 2.4. Biosensor Preparation, Electrochemical Cell and Voltammetric Parameters

The PPO-*Cordia superba* electrochemical biosensor was prepared in agreement with previous data [3] with 70 mg graphite powder and 50 µL of *Cordia superba* extract. The mixture was allowed to dry at room temperature for approximately 2 h (28 °C). Then, 30 mg of mineral oil was added to obtain a homogeneous paste. A quantity of the agglutinated paste was used to fill a cavity of 2 mm in diameter and 0.5 mm deep in the electrode holder, originating the PPO electrochemical biosensors based on crude extract of *Cordia superba,* CP50*CS.*

All voltammetric analyses were performed on a PGSTAT^®^ model 204 potentiostat/galvanostat integrated with the NOVA2 software 1^®^ (Metrohm Autolab, Utrecht, The Netherlands). The experiments were carried out in an electrochemical cell (10 mL) with a system of three electrodes consisting of PPO electrochemical biosensor, a Pt wire and Ag/AgCl/KClsat as working, auxiliary and reference electrode, respectively. The carbon paste was manually renewed for each experiment, in order to ensure the results’ effectiveness and reproducibility.

Based on previous results [3,15], the PPO electrochemical biosensor was always conditioned before use in acetate buffer (pH 5.0), where, using cyclic voltammetry, 10 successive scans, 50 mV s^−1^ were performed, in a potential range of 0.0 to 1.0 V. Differential pulse voltammetry (DPV) technique was chosen to perform the analyses of phenolic compounds. The operating conditions were pulse amplitude of 50 mV, pulse width of 0.5 s and scan rate of 10 mV s^−1^. All the electrochemical experiments presented within this work were carried out in acetate buffer at pH 5.0, in triplicate (n = 3) and at room temperature (28 ± 2 °C). 

### 2.5. Antioxidant Activity Determinations

The measurement of the organic radical scavenging activity was carried out according to the DPPH (2,2-diphenyl-1-picryl-hydrazyl-hydrate) method [16,17], which is based on the capture of the DPPH radical by antioxidants, producing a decrease in absorbance at 515 nm. The reaction mixture consisted of adding 0.3 mL of sample and 2.7 mL of the DPPH radical, leaving it in a dark environment for 5 min and performing the reaction reading, using the blank with methyl alcohol as a reference. The quantification of phenolic compounds in the kombucha samples was performed in triplicate and expressed by equivalent of the DPPH standard curve. 

The Folin-Ciocalteu (FC) spectrophotometric method [18] was used to determine total phenolic compounds in kombucha. Each aliquot of 50 µL of kombucha sample at a concentration of 1% was placed in a test tube containing 1 mL of distilled water and 250 µL of the FC reagent. After 5 min, 750 µL of a 20% Na_2_CO_3_ solution and 2950 µL of distilled water were added. The mixture was incubated in the absence of light for 60 min; afterwards, the absorbance was measured in a spectrophotometer at 765 nm, using the blank solution as a reference. The quantification of phenolic compounds in kombucha samples was carried out in triplicate and expressed by means of gallic acid equivalents in µM, from a calibration curve obtained under the same conditions for sample analysis. 

Absorption spectra were recorded using an Agilent 8453 UV–Vis spectrophotometer with a photodiode array detector and UV-Visible ChemStation software (Agilent Technologies, Inc., Santa Clara, CA, USA).

### 2.6. Statistics

In order to test the difference between analyzed groups, one-way ANOVA was performed with 0.05 significance level (H0/null hypothesis: means comparison was equal between tested groups). The data analyzed was the analytical signal of the enzymatic extract of *Cordia superba* different plant parts (CP/control, ripe fruit, green fruit, leaves and branches), the analytical signal of the tested phenolic markers (Cafeic acid, catechin, catechol, gallic acid, epicatechin and rutin) and the modified sensor’s performance (CP/control, *M. colocasiae* and *C. superba*). The test was performed in Matlab R2022a.

## 3. Results and Discussion

**Figure 1:**
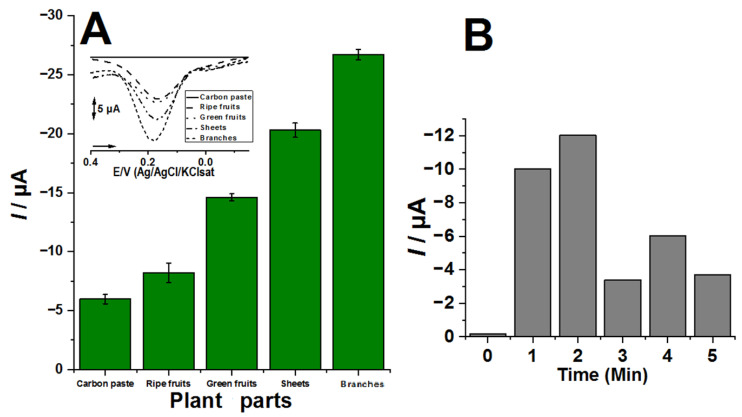
(**A**) Electrochemical CP50CS biosensors prepared from the crude extract obtained from different parts of *Cordia Superba* and their electrochemical responses vs. 10 μM catechin solution in acetate buffer pH 5.0. Insert: voltammograms of plant parts of *Cordia superba* (**B**) Different enzyme reaction times of the CP50CS biosensors in the 10 μM catechin, pH 5.0, before electrochemical analysis.

In order to investigate the PPO electrochemical biosensor proposed here, it was prepared based on previous results from 70 mg of CP, 50 µL of *Cordia Superba* (*CS*) extract and 30 mg of mineral oil and called CP50*CS* electrochemical biosensor. CP50*CS* biosensors were then prepared from the crude extract obtained from different parts of *Cordia Superba* and their electrochemical responses were evolved against a 10 μM catechol solution for 2 min in acetate buffer of pH 5.0 [7], as shown in Figure 1. The cathodic peaks of the CP50*CS* biosensors were then recorded by DPV [7].

Statistical difference was observed between all tested groups in Figure 1, with a 0.05 significance level. The aliquot of plant material that presented the best results were the branches, thus being selected to analyze the enzymatic reaction times of the biosensor with the substrate, in order to promote biochemical oxidation and subsequently electrochemical reduction, as shown in Figure 1B.

Thus, the CP50*CS* electrochemical biosensor was optimized, having been prepared with the *CS* branch extract and an interaction time with the substrate of 2 min, as shown in Figure 1B. The interaction time of the sensor with the substrate is important as it establishes the time of action of PPO on the oxidation of catechol moiety by O_2_, as well as the response of the electroreduction of *o*-quinone [5,6].

**Figure 2:**
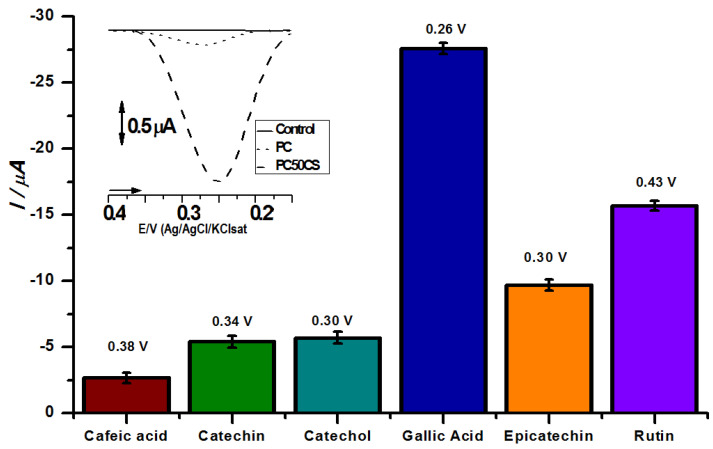
Analysis of phenolic compounds at 10 μM by CP50CS electrochemical biosensor in acetate buffer pH 5.0 after 2 min of enzymatic reaction. Insert: Comparison of the control, carbon paste (CP) and the CP50CS biosensor versus 10 μM catechol.

The response of the CP50*CS* biosensor proposed in this work was also investigated for the detection of other phenolic compounds, caffeic acid, epicatechin, catechol, gallic acid and rutin, as shown in Figure 2. As expected, the prepared CP50*CS* sensors showed high cathodic currents for all chemical species investigated, between 2.5 and 25 µA. The highest sensitivity of the sensor was observed for gallic acid, rutin, epicatechin and catechol, respectively, as shown in Figure 2.

The chemical and physicochemical properties of the phenolic compounds investigated here such as solubility, aromaticity, reactivity, pkas values, oxidation potentials and oxidation by-products, as expected, considerably influence their interactions with the CP50*CS* electrochemical biosensor [3,5,6,7,19]. All phenolic antioxidants with the exception of cafeic acid had a current response above 5 uA. Gallic acid and rutin, with 4 and 9 hydroxyl groups, respectively, showed higher peak currents in relation to the sensor when compared with the other phenolic compounds, as shown in Figure 2. Catechol has 2 hydroxyls and is widely used as the main standard in antioxidant activities. Caffeic acid has 2 hydroxyls and presented a less satisfactory result than the other standards in relation to the enzymatic interaction, but superior to the carbon paste without modification. Statistical difference was observed between all tested groups in Figure 2, with the exception of catechin and catechol (which were statistically equal) with a 0.05 significance level.

**Figure 3:**
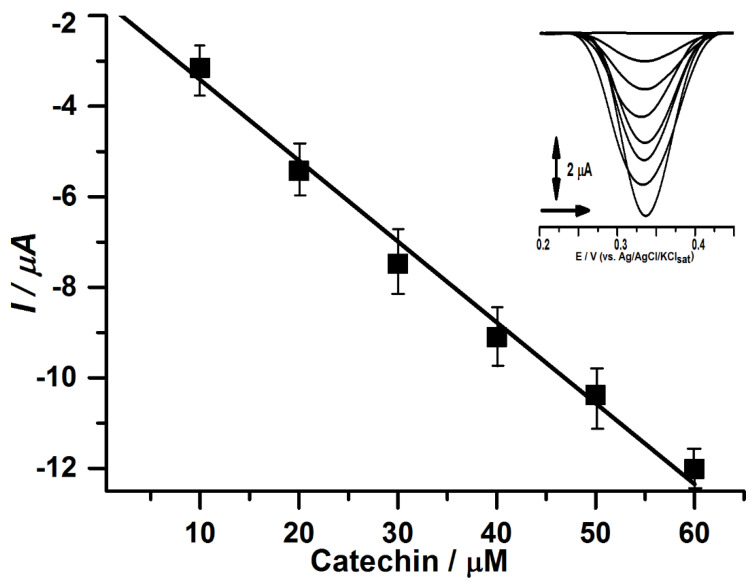
Calibration curve of catechin from 10.00 to 60.00 µM in acetate buffer pH 5.0, using CP50*CS* electrochemical biosensor, after 2 min of enzymatic reaction.

A calibration curve (Figure 3) was developed based on the response presented to the biosensor; the catechin compound was used due to the majority presence in kombucha samples, in order to assess whether the proposed method presented a linear response to the increase in the concentration of analysis. Figure 3. Calibration curve of catechin from 10.00 to 60.00 µM in acetate buffer pH 5.0, using CP50*CS* electrochemical biosensor, after 2 min of enzymatic reaction.

The proposed methodology for the limit of quantification and limit of detection uses the value of the standard deviation of the intercept to calculate the limit of detection, instead LQ and LD can be achieved by 3 and 10 times the blank measured values, respectively. Prescription 1 was used to obtain LD, which will give a signal equal to three times the baseline noise level, calculated in electrolyte with prescription.

**LD = 3SB/b** (1)

The LQ also evaluated in this work is defined as the lowest value determined for the proposed methodology, where we consider that the equipment limit has not yet been reached and corresponds to 99%, given by the relationship in Equation (2).

**LQ = 10SB/b** (2)

The calibration curve exhibited a significant linear segment in the range from 10.00 to 60.00 µM (R^2^ = 0.9961) (Figure 3) with a detection limit (LOD) of 0.13 µM (3σ/S, where σ is the standard deviation of the blank signal for n = 3 and S the sensitivity) and the limit of quantification (LOQ) of 0.39 µM (10σ/S, where σ is the standard deviation of the blank signal for n = 3 and S the sensitivity). The results obtained in this work were compared to similar surveys displayed in Table 1.

Compared to works previously realized by the research group with the fungus *Marasmiellus colocasiae* [3], the *Cordia superba* biosensor showed superior results in terms of LOD and LOQ, being more selective and specific in the face of the biochemical reaction in obtaining analytical signal [5,6].

**Figure 4:**
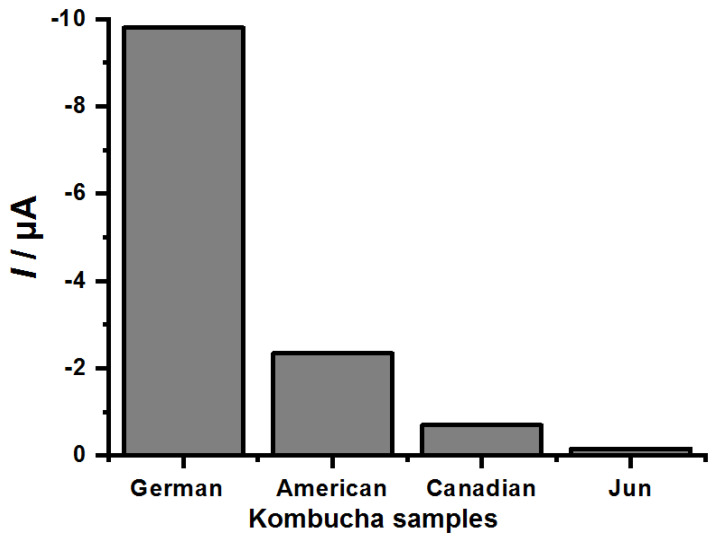
Analysis of kombucha samples from different backgrounds with CP-50EP in acetate buffer pH 5.0 after 2 min of enzymatic reaction.

Kombucha samples were made at the Faculty of Pharmacy of the Federal University of Goiás, with commercial green tea and using SCOBYs from different origins, from German, American, Canadian or the Jun SCOBY, attributing different flavors, acidity levels and antioxidant activities. The antioxidant activity (phenolic compounds) of the kombucha samples was then analyzed using the CP50*CS* electrochemical biosensor, where they were exposed for 2 min to the samples, as shown in Figure 4. Subsequently, the DP voltammograms of these sensors were recorded in the potential range from 0.20 to 0.45 V in acetate buffer pH 5.0, as shown in Figure 4. Statistical difference was observed between all tested groups in Figure 4, with a 0.05 significance level, showing that the modification significantly improved the performance of the analysis.

The fermented products with German SCOBY content presented a vinegar with greater acidity, a content derived from the preparation with saccharide, as well as giving it the highest cathodic peak current and thus antioxidant activity, as shown in Figure 4. The kombucha samples analyzed with CP50*CS* biosensor that showed lower antioxidant activities were those fermented with Canadian and Jun SCOBY, as shown in Figure 4 and Table 2, in agreement with previous results [23,24,25,26].

Biosensors have peculiarities that make biological components unique, such as LOD, LOQ, sensitivity and interactions, exploring different ways of detecting and potentiating the transduction and visualization of the analytical signal.

DPPH and Folin-Ciocalteu spectrophotometric assays were used for comparison with the proposed electrochemical method, as shown in Table 2. The DPPH free radical method is an antioxidant assay based on electron transfer that produces a violet solution in ethanol [16,17]. The Folin–Ciocalteu is a redox reagent composed by phosphomolybdate and phosphotungstate that in presence of phenolic antioxidants undergo reduction yielding a blue colour, that is spectrometrically measured at 765 nm wavelength [3,27].

The CP50*CS* electrochemical biosensor proposed here was used to quantify the antioxidant activity (total phenolic compounds) of the kombucha samples and the results were very similar to those obtained with the DPPH and Folin-Ciocalteu spectrophotometric methods, as shown in Table 2. All results indicated greater antioxidant activity from kombucha samples prepared with Scoby from Germany. These results thus demonstrate the excellent performance and applicability of the CP50*CS* sensor for the quantification of antioxidant activity in real samples. However, a greater number and variety of samples are still needed. 

## 4. Conclusions

Studies that corroborated with the literature showed that the enzymatic biosensor of *C. superba* showed relevant results for the proposal of a new analytical tool with low cost, high sensitivity and reproducibility, with real food samples under usual conditions for the detection of phenolic compounds, optimizing the development of electrochemical detection devices for food quality control.

This work sets precedents for the implementation of new technologies, such as miniaturization through printed electrodes and real-time analysis, optimizing analyzes in quality control of kombucha and other fermented beverages in the future.

The biosensor made with the crude plant extract of *C. superba* was satisfactory for the detection of phytomarkers and catechin quality control of kombucha samples based on *Cammelia sinensis.*

Factors such as detection, quantification, precision, accuracy and stability presented relevant results due to the good analytical response of the biochemical sensor. The ease of obtaining the plant enzymatic extract, as well as the low cost and fast analysis, are attractive for an alternative quality control device for food.

## Figures and Tables

**Table 1:** Comparison of the results obtained using the CP50*CS* biosensor developed in this research with other immobilized electrodes of PPO for catechin/catechol detection.

Enzyme Source	Method	Linear Range (µM)	Limit of Detection (µM)	Reference
*C. superba*	DPV	30–300	0.13	This work
*M. colocosiae*	DPV	50–300	0.17	[3]
*Jenipapo fruit* (*Genipa americana* L.)	DPV	10–310	7	[18]
Mushrooms	Amperometric	0.5-24.0	0.3	[19]
*Manilkara Z.* (sapota) Fruit	Chrono amperometric	1.0–15.0	0.47	[20]
*Agaricus bisporus*	Amperometric	0.5–101	0.15	[21]
Purchased from Sigma	Cyclic voltammetry	1.0–100	0.01	[22]

**Table 2:** Analysis of kombucha samples with CP50*CS* biosensor by DPV, in acetate buffer pH 5.0 after 2 min of enzymatic reaction; DPPH and Folin-Ciocalteu spectrophotometric assays.

Samples	E_pc_ (V) (n = 3)	I_pc_ (µA) ± Standard Deviation (n = 3)	DPPH Catechin Equivalent (µM) ± Standard Deviation (n = 3)	Folin-Ciocalteu Catechin Equivalent (µM) ± Standard Deviation (n = 3)
Germany	0.35	−9.8 ± 0.38	−9.8 ± 0.0095	−2.7 ± 0.02
American	0.35	−2.34 ± 0.42	−0.12 ± 0.055	−2.4 ± 0.01
Canadian	0.34	−0.7 ± 0.44	−5.08 ± 0.0085	−2.1 ± 0.11
Jun (honey)	0.34	−0.16 ± 0.38	−0.14 ± 0.006	−2.06 ± 0.09

## Data Availability

No new data were created or analyzed in this study. Data sharing is not applicable to this article.
